# Social cohesion and belonging predict the well-being of community-dwelling older people

**DOI:** 10.1186/s12877-015-0027-y

**Published:** 2015-03-21

**Authors:** Jane M Cramm, Anna P Nieboer

**Affiliations:** Institute of Health Policy and Management, Erasmus University Rotterdam, Burgemeester Oudlaan 50, Rotterdam, 3000 DR the Netherlands

**Keywords:** Neighborhood social deprivation, Social production function, Community, Social well-being, Physical well-being

## Abstract

**Background:**

The neighborhood social environment has been identified as an important aspect of older people’s well-being. Poor neighborhood conditions can pose difficulties in obtaining support, especially for older people who live alone. Although social environments have been found to be related to well-being among older people, the longitudinal relationship between the social environment and well-being remains poorly undestood. Research on the effects of changes in neighborhood characteristics, such as social cohesion and social belonging, on well-being is lacking. Therefore, the study aims are (i) describe social cohesion, social belonging, and instrumental goals to achieve well-being among community-dwelling older people, (ii) determine whether these factors varied according to neighborhood social deprivation and compare these findings to those from chronically ill/previously hospitalized older people, and (iii) identify longitudinal relationships between social cohesion and belonging and well-being.

**Methods:**

Independently living Dutch older adults (aged ≥ 70 years) were asked to complete questionnaires in 2011 (T0) and 2013 (T1). Response rates at T0 and T1 were 66% (945/1440) and 62% (588/945), respectively. Descriptive statistics, paired sample *t*-tests, analysis of variance, univariate analyses and multilevel regression analyses controlling for background characteristics and baseline well-being were performed.

**Results:**

Of 945 respondents [43% male; mean age, 77.5 ± 5.8 (range, 70–101) years], 34.7% were married and 83.3% were Dutch natives. Social cohesion remained constant over time, whereas social belonging improved (*p* ≤ 0.05). Older people living in socially deprived neighborhoods report poorer overall well-being and instrumental goals to achieve well-being. Baseline social cohesion, changes therein (both *p* ≤ .001), baseline social belonging, and changes therein (both *p* ≤ .05) predicted well-being at T1.

**Conclusion:**

This study showed that social cohesion, belonging, and changes therein predict the social as well as physical well-being of community-dwelling older people in the Netherlands over time. The creation of stronger ties among neighbors and a sense of belonging is needed.

## Background

With ongoing increases in life expectancy, worldwide populations are aging [1]. This progress also poses the challenge of maintaining older people’s well-being. As co-producers of a sustainable health and social care system, older people should be supported in managing their well-being and social environments [2]. Continual community engagement, such as by helping neighbors and/or being involved in neighborhood decisions, may allow older people to realize a lifelong potential for well-being.

**Figure 1:**
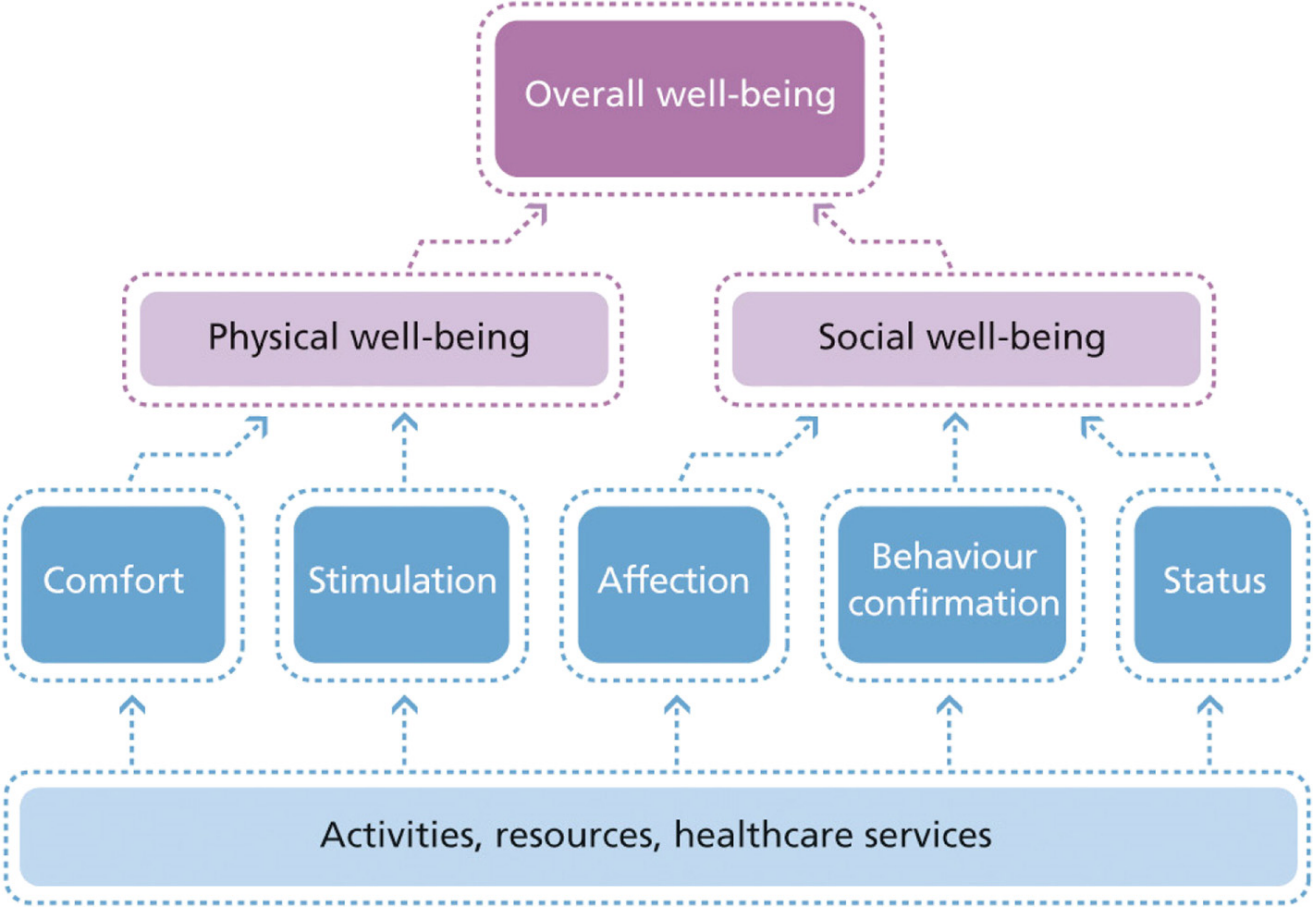
**Social production function theory explaining the hierarchy of well-being.**

The concept of well-being encompasses an individual’s overall perceived quality of life [3,4], and is thus a broader measure than health status. Individuals’ ability to achieve well-being can be assessed with great specificity through social production function (SPF) theory. This theory asserts that people produce their own well-being by trying to optimize the achievement of instrumental goals (stimulation, comfort, status, behavioral confirmation, and affection) that provide the means to achieve the larger, universal goals of physical and social well-being (Figure 1) [5,6]. People will generally strive to achieve physical and social well-being within the available set of resources and constraints.

*Physical well-being* is achieved through the instrumental goals of stimulation [activities producing arousal, e.g., mental and sensory stimulation, physical effort and (competitive) sports–although prolonged physical effort can become unpleasant] [2,5-7] and comfort (absence of deleterious stimuli, such as fear, pain, hunger, and thirst). Stimulation (within the pleasant range) and comfort are each related to physical well-being by a monotonically increasing production function with decreasing marginal product. Thus, an additional unit of comfort or stimulation becomes less valuable with increasing physical well-being. *Social well-being* is achieved by realizing the three instrumental goals of affection [receiving love for who one is as a person, regardless of one’s assets or actions (e.g., friendship, intimacy, and emotional support given by one’s partner, children, or other loved ones)], behavioral confirmation (feeling of doing the “right thing” in the eyes of relevant others, even without direct reinforcement), and status (social ranking based on, e.g., one’s profession, lifestyle, or specific talents and social approval of one’s accomplishments) [2,6,7]. These instrumental goals are related to social well-being in the same way described for physical well-being, with reduced value for individuals with high levels of social well-being.

Given that people actively seek ways to maintain or improve their physical and social well-being, they will be resourceful and find substitutions for losses when possible [8]. Physical and social well-being are general goals within a hierarchy with the ultimate goal of subjective well-being [2]. Frameworks such as that provided by SPF theory facilitate a fuller understanding of older people’s well-being and how it may be enhanced through consideration of the social environment, as well as individual characteristics [9-11].

The neighborhood social environment has been identified as an important aspect of older people’s well-being [9]. This environment can positively affect morbidity and mortality, and aging individuals’ increased dependency can be met with the establishment and maintenance of supportive relationships in a socially cohesive network of neighborhood resources [12-14]. Conversely, poor neighborhood conditions can pose difficulties in obtaining support, especially for older people who live alone [15]. Because they spend a greater proportion of their lives in their neighborhoods [16,17], neighborhood environments are critical elements of support systems for older people whose declining health results in frailty and/or social isolation, as well as those with limited mobility, finances, and/or access to transportation [18,19].

The examination of neighborhood social cohesion involves the assessment of levels of affective and instrumental support (trust, reciprocity, and social bonds) provided within the neighborhood environment [20-23]. An individual’s sense of belonging in a neighborhood depends on a multitude of subjective and objective factors, including his or her physical and psychological status and conception of the meanings of “neighborhood” and “neighboring,” as well as local environmental characteristics [24]. Social cohesion positively impacts the strength of relationships and social participation in, as well as collective attachment to, the neighborhood, and is thus expected to enhance individuals’ well-being [25]. In contrast, a lack of cohesion can generate social disorder, conflict, and extreme inequality, with little interaction within and among communities and little attachment to place [26].

The availability of opportunities to realize affection, status, comfort, and stimulation varies widely among neighborhoods [27]. Older persons may have particular difficulty in realizing the social goals of affection, behavioral confirmation, and status in socially deprived neighborhoods. The physical goals of comfort and stimulation may also be compromised as a result of a lack of support in times of need or feeling unsafe in the neighborhood. The importance of supporting older people in neighborhoods in realizing these instrumental goals to achieve well-being is even more evident in light of research showing that loss of comfort and affection are the main predictors of dependent living [28]. Steverink [28] argued that status is likely the first instrumental goal to be compromised after retirement, given the loss of individuals’ and/or their partners’ occupational and social positions. As a consequence, older people’s realization of social well-being depends increasingly on behavioral confirmation and affection. However, behavioral confirmation (which requires the ability to play multiple roles) can be difficult to achieve in the face of age-related physical limitations. Thus, affection is the primary means of achieving social well-being for older people. Physical limitations (e.g., difficulty or inability to travel or even to leave the house) also reduce the opportunity for stimulation, rendering comfort an increasingly important means of achieving physical well-being as people age.

Although social environments have been found to be related to well-being among older people [9,29], the longitudinal relationship between these factors remains poorly understood. Research on the effects of changes in neighborhood characteristics, such as social cohesion and social belonging, on (instrumental goals to achieve) well-being is lacking. Such investigations would be of particular importance in determining whether positive changes in neighborhood social environments improve the well-being outcomes of community-dwelling older adults and, conversely, whether deterioration of these environments is detrimental to older adults’ well-being. By distinguishing different levels of goals with regard to well-being and by realizing that lower-level goals are needed to achieve higher-level goals, we can trace the consequences of living in poor neighborhoods in term of social cohesion and belonging for the well-being of community-dwelling older people, and thereby determine what changes are needed to protect their well-being. Since people achieve physical and social well-being within the set of resources and constraints they face [6] it may be more difficult for some (e.g. those living in a socially deprived area, those dealing with a chronic condition or being hospitalized) to achieve a certain degree of well-being. We expect that dealing with the consequences of a chronic condition or hospitalization comes with greater difficulties in maintaining ones physical well-being, while living in a socially deprived neighborhood is expected to complicate maintaining a certain degree of social well-being. To examine these issues, this study aimed to (i) describe social cohesion, social belonging, and instrumental goals to achieve well-being among community-dwelling older people; (ii) identify variation in these factors according to the degree of neighborhood social deprivation and compare these findings to those from chronically ill older people and older people after hospitalization; and (iii) identify longitudinal relationships between (changes in) social cohesion and belonging and well-being.

## Methods

### Participants

A sample of 1440 independently living older adults in four districts of Rotterdam (Lage Land/Prinsenland, Lombardijen, Oude Westen, and Vreewijk) was randomly identified using the population register. The target population of the sample was defined as individuals ≥ 70 years who speak Dutch and do not live abroad or in an institution during the field-work period. The sample included about 430 eligible older adults per district and was proportionate to the 72 neighborhoods in these districts and to age groups (70–74, 75–79, 80–84, and ≥85 years). Eligible older adults were asked by mail to complete a written or online questionnaire in 2011 (T0). Respondents were rewarded with a 1/5 ticket in the monthly Dutch State Lottery. Non-respondents were first sent a reminder by mail, were then asked by telephone to participate, and were finally visited at home if they could not be reached by telephone. Two years later (in 2013; T1), respondents to the 2011 questionnaire were asked again to fill in a questionnaire using the same strategy. The ethics committee of Erasmus University Medical Centre of Rotterdam approved this study (MEC-2011-197) and all respondents provided informed consent. This study took place in the context of a larger evaluation of a transition experiment aiming to facilitate independently-living frail elderly persons (70+) to live the life they wish to live, improving their well-being. The transition experiment started two Rotterdam districts (Lage Land/Prinsenland and Lombardijen) and was later extended to the Oude Westen and Vreewijk districts. The larger evaluation consisted of an inventory (this study) as well as an experiment with a controlled pre-post measurement (including only 185 older people). The inventory is taken among the elderly (70+) in the four districts of Lage Land/Prinsenland, Lombardijen, Oude Westen, and Vreewijk to investigate the general situation of elderly in these districts where the experiment takes place. A detailed description of the study design can be found in the study protocol [30].

### Measures

Well-being was measured with the 15-item version of the Social Production Function Instrument for the Level of Well-Being (SPF-IL; Appendix) [7]. This scale measures levels of physical (comfort, stimulation) and social (behavioral confirmation, affection, status) well-being. Examples of questions are: “Do people pay attention to you?” (affection), “Do you feel useful to others?” (behavioral confirmation), “Are you known for the things you have accomplished?” (status), “In the past few months have you felt physically comfortable?” (comfort), and “Do you really enjoy your activities?” (stimulation). Answers were given on a four-point scale ranging from never (1) to always (4), with higher mean scores indicating greater well-being. Cronbach’s alpha values of the SPF-IL were 0.86 at T0 and 0.88 at T1, indicating good reliability. The reliability of this instrument for the assessment of well-being in older populations has been proven [9,29,31].

The main explanatory variables in this study were social cohesion and social belonging in the neighborhood. The study questionnaire thus incorporated the neighborhood social cohesion and social belonging scales [32,33]. These scales have been used in studies conducted in the UK, which have provided evidence of their validity and reliability [34,35]. The eight-item social cohesion instrument and seven-item social belonging instrument are structured by a five-category Likert response scale ranging from “strongly agree” to “strongly disagree” [18,30]. Possible scores on the social cohesion and belonging scales range from 8 to 40 and 7 to 35, respectively with higher scores indication higher levels of social cohesion and belonging. Cronbach’s alpha values of the social cohesion instrument in the current study were 0.75 at T0 and 0.79 at T1, and those of the social belonging instrument were 0.84 at T0 and 0.86 at T1.

The questionnaire also solicited information about respondents’ gender, age, marital status, ethnic background, and education. The highest educational qualification achieved was measured using a seven-point scale ranging from 1 (primary school or less) to 7 (university degree). Education was dichotomized as poor (1; primary school or less) or good (0; more than primary school).

### Statistical analyses

First, we employed descriptive statistics to characterize the study population. Second, we selected the 10% worst neighborhoods based on the sum scores of the social cohesion and social belonging scales. We described instrumental goals to achieve well-being (stimulation, comfort, status, behavioral confirmation, and affection), social cohesion, and social belonging within these poor neighborhoods and compared them to the overall average. Third, we investigated changes in well-being and social cohesion and belonging over time using paired-sample *t*-tests. Furthermore, social cohesion and belonging were compared among neighborhoods using analysis of variance. Fourth, univariate analyses were performed to determine associations between background characteristics, social cohesion, social belonging and well-being among community-dwelling older adults. Finally, multilevel analyses were employed to investigate whether (changes in) social cohesion and belonging predicted well-being while controlling for well-being at T0 and background characteristics (age, gender, marital status, education level, and being born in the Netherlands). Two-sided tests were used to determine significance (*p* ≤ .05) in all analyses. All statistical analyses were conducted using SPSS software (ver. 19.0; IBM).

## Results

Our final sample at T0 consisted of 945 respondents (66% response rate). Of the 945 respondents participating in this study at T0, 43% were men. Their average age was 77.5 (range, 70–101; standard deviation, 5.8) years. About one-third (34.7%) of respondents were married and 83.3% were born in the Netherlands (Table 1). These figures are comparable to the mean percentage of males (43%) and being born in the Netherlands (87%) among older people (aged ≥ 70 years) in the Dutch population in 2013 [36].

**Table 1:** **Baseline characteristics of community-dwelling older adults**

	**Respondents (** ***n*** **= 945)**
Mean age (years)	77.48 ± 5.78 (70–101)
Gender (male)	43%
Marital status (married)	35%
Low educational level	22%
Born in the Netherlands	83%
Well-being (SPF-IL)	2.56 ± 0.45 (1–4)
Social cohesion	24.39 ± 5.38 (8–39)
Social belonging	26.21 ± 5.01 (7–35)

Notes: Data are expressed as mean ± standard deviation (range) or percentage.

SPF-IL, Social Production Function Instrument for the Level of Well-Being.

Response rate at T1 was 62% (*n* = 588). We compared baseline characteristics of the 588 participants who completed both questionnaires to those who completed T0 only. No difference in social cohesion, social belonging, gender, or marital status was found. On average, respondents who completed both questionnaires were younger (77.11 ± 5.33 vs. 78.07 ± 6.41 years; p < .05), reported better well-being (2.60 ± 0.54 vs. 2.50 ± 0.53; p < .01), were more often born in the Netherlands (85.2% vs. 80.1%; p < .05) and less often lower educated (19.6% vs. 26.3%; p < .05) than those who completed only one questionnaire.

We also compared baseline characteristics of participants born in the Netherlands (83%) compared to those born in a different country (17%). On average, respondents born in the Netherlands were older (77.88 ± 5.86 vs. 75.46 ± 4.49 years; p < .001), reported better well-being (2.58 ± 0.54 vs. 2.45 ± 0.52; p < .01), better social cohesion (24.63 ± 5.53 vs. 23.20 ± 4.98; p < .01) and better social belonging (26.37 ± 4.99 vs. 25.44 ± 5.05; p < .05) than those not born in the Netherlands. Furthermore, respondents not born in the Netherlands were more often lower educated (42.4% vs. 18.0%; p < .001), married (41.8% vs. 33.3%; p < .05) and male (57.0% vs. 40.2%; p < .001).

Analyses of variance showed that social cohesion and belonging differed among neighborhoods at T0 and T1 (all *p* ≤ .05). Social cohesion and belonging scores ranged from 19.25 to 32.00 and 21.13 to 30.33, respectively.

Table 2 displays percentages of respondents’ disagreement with social cohesion and social belonging items. Comparison of responses from older people living in the 10% worst neighborhoods with overall averages obviously showed poorer outcomes but additionally showed a great degree of variation on most social cohesion and belonging items. More than 90% of respondents from socially deprived neighborhoods did not visit or borrow/exchange things with their neighbors. The majority of respondents disagreed with the statements that living in their neighborhood gave them a sense of community (84%), that they could turn to someone in the neighborhood when they needed advice (80%), that their friendships and associations with other people in the neighborhood mean a lot to them (74%), and that they were willing to work together with others to improve their neighborhood (70%). Only 10% of all respondents, but 40% of those living in socially deprived neighborhoods, disagreed with the statement that they believed their neighbors would help in an emergency.

**Table 2:** **Social cohesion and belonging by neighborhood social deprivation**

	**10% worst neighborhoods**	**All neighborhoods**
	Disagree (%)	Disagree (%)
*Social cohesion*		
I visit my neighbors in their homes.	91%	51%
The friendships and associations I have with other people in my neighborhood mean a lot to me.	74%	25%
If I needed advice about something, I could go to someone in my neighborhood.	80%	27%
I believe my neighbors would help in an emergency.	40%	10%
I borrow things and exchange favors with my neighbors.	92%	61%
I would be willing to work together with others on something to improve my neighborhood.	70%	48%
I rarely have a neighbor over to my house to visit.	36%	39%
I regularly stop and talk with people in my neighborhood.	56%	22%
*Social belonging*		
Overall, I am attracted to living in this neighborhood.	33%	6%
I feel like I belong to this neighborhood.	50%	8%
Given the opportunity, I would like to move out of this neighborhood.	32%	76%
I plan to remain a resident of this neighborhood for a number of years.	40%	9%
I like to think of myself as similar to the people who live in this neighborhood.	56%	14%
Living in this neighborhood gives me a sense of community.	84%	26%
Overall, I think this is a good place to bring up children.	64%	23%

We investigated changes in well-being, social cohesion and social belonging over time. Paired-sample *t*-tests showed that social cohesion remained fairly constant over time (24.63 at T0 *vs*. 24.42 at T1; *p* = .29; *n* = 561), while respondents’ sense of social belonging improved significantly (26.32 at T0 *vs*. 26.73 at T1; *p* ≤ .05; *n* = 558). No change in overall mean well-being (2.61 at T0 *vs*. 2.59 at T1; *p* = .28; *n* = 552), affection (2.62 at T0 *vs*. 2.67 at T1; *p* = .16; *n* = 557), status (1.96 at T0 *vs*. 1.91 at T1; *p* = .14; *n* = 517), or comfort (2.72 at T0 *vs*. 2.70 at T1; *p* = .48; *n* = 557) was found. However, as expected, we found that behavioral confirmation (2.91 at T0 *vs*. 2.83 at T1; *p* ≤ .01; *n* = 548) and stimulation (2.83 at T0 *vs*. 2.76 at T1; *p* ≤ .01; *n* = 571) decreased significantly over time.

Scores for overall well-being and the instrumental goals needed to achieve it were significantly lower in socially deprived neighborhoods than in the overall sample. In addition, social well-being was significantly worse among respondents living in socially deprived neighborhoods than among Dutch chronically ill patients and those who had recently been hospitalized (aged ≥ 70 years); physical well-being was worse than that of chronically ill patients and comparable to that of recently hospitalized older people. Status did not decline over time in our sample, but respondents (especially those living in socially deprived areas) had very low status compared with the other samples (Table 3).

**Table 3:** **Well-being and instrumental goals to achieve well-being in adults aged ≥ 70 years**

	**10% worst neighborhoods (this study; 2011)** ***n*** **= 97**	**All neighborhoods (this study; 2011)** ***n*** **= 945**	**Chronically ill (2011)** ***n*** **= 928**	**After hospitalization (2010/2011)** ***n*** **= 219**
*Social well-being*				
Affection	1.88 (.70)	2.63 (.82)^***^	3.10 (.68)^***^	2.82 (.44)^***^
Behavioral confirmation	2.27 (.76)	2.86 (.73)^***^	3.08 (.60)^***^	3.31 (.61)^***^
Status	1.57 (.64)	1.90 (.75)^***^	2.17 (.61)^***^	3.22 (.53)^***^
*Physical well-being*				
Comfort	2.28 (.80)	2.63 (.83)^***^	2.55 (.73)^***^	2.19 (.72)
Stimulation	2.47 (.75)	2.77 (.75)^***^	2.90 (.65)^***^	2.54 (.78)
*Overall well-being*	2.08 (.50)	2.56 (.54)^***^	2.76 (.44)^***^	2.78 (.62)^***^

Notes: Data are expressed as mean (standard deviation).

^***^ *p* ≤ .001 *vs*. 10% worst neighborhoods (two-tailed *t*-test).

Univariate analyses showed that gender and educational level at T0 (both *p* ≤ .01), being born in the Netherlands (*p* ≤ .05), and social cohesion and belonging at T0 and T1 (all *p* ≤ .001) were significantly related to the well-being of older adults at T1 (Table 4). Being born in the Netherlands, social cohesion, and belonging positively affected the well-being of older people at T1, whereas negative relationships were found with low educational level and male gender. No significant relationship was found between well-being at T1 and age or marital status at T0.

**Table 4:** **Associations among individual characteristics, social cohesion, social belonging, and well-being of older adults**

	**1**	**2**	**3**	**4**	**5**	**6**	**7**	**8**	**9**	**10**
1. Age at T0										
2. Gender (male) at T0	-.17^***^									
3. Marital status (married) at T0	-.28^***^	.37^***^								
4. Low educational level at T0	.02	-.04	-.05							
5. Born in the Netherlands	.16^***^	-.13^***^	-.07^*^	-.22^***^						
6. Social cohesion at T0	-.07^*^	-.03	.02	-.08^*^	.10^**^					
7. Social cohesion at T1	-.11^**^	-.03	.12^**^	-.08	.08^*^	.66^***^				
8. Social belonging at T0	.06	-.05	.01	-.04	.07^*^	.45^***^	.34^***^			
9. Social belonging at T1	.03	-.04	.03	-.03	.02	.35^***^	.48^***^	.61^***^		
10. Well-being at T0	-.04	-.04	.05	-.08^*^	.09^**^	.45^***^	.42^***^	.37^***^	.32^***^	
11. Well-being at T1	-.05	-.12^**^	.05	-.13^**^	.08^*^	.40^***^	.47^***^	.32^***^	.34^***^	.69^***^

Notes: T0, 2011; T1, 2013.

^***^ *p* ≤ .001, ^**^ *p* ≤ .01, ^*^ *p* ≤ .05 (two-tailed).

Multilevel regression analyses that controlled for older adults’ background characteristics and well-being at T0 showed that social cohesion at T0 and changes therein (both *p* ≤ .001), and social belonging at T0 and changes therein (both *p* ≤ .05) predicted well-being at T1 (Table 5). Poor educational level was a negative predictor and male gender was a positive predictor of community-dwelling older adults’ well-being. Although univariate analyses showed positive relationships between being born in the Netherlands and well-being at T0 and T1, these associations were not significant in multivariate analyses.

**Table 5:** **Predictors of well-being at T1 (2013), as assessed by multilevel random-intercepts regression analyses (** ***n*** **= 532)**

	***β***	**SE**
Constant	2.54^***^	.02
Well-being at T0	.33^***^	.02
Age	-.02	.02
Gender (male)	.05^**^	.02
Marital status (married)	.00	.02
Low educational level	-.05^**^	.02
Born in the Netherlands	-.01	.02
Social cohesion at T0	.07^***^	.02
Changes in social cohesion (T1 – T0)	.09^***^	.02
Social belonging at T0	.05^*^	.02
Changes in social belonging (T1 – T0)	.04^*^	.02

Notes: These findings are based on data from respondents who filled in questionnaires at both T0 and T1 (*n* = 588). Listwise deletion of missing cases led to a final sample of 532 respondents.

SE, standard error; T0, 2011; T1, 2013.

^***^ *p* ≤ .001, ^**^ *p* ≤ .01, ^*^ *p* ≤ .05 (two-tailed).

## Discussion

The neighborhood social environment has been identified as an important aspect of older people’s well-being. Poor neighborhood conditions can pose difficulties in obtaining support, especially for older people who live alone since they spend a greater proportion of their lives in their neighborhoods. Research on the effects of changes in neighborhood characteristics, such as social cohesion and social belonging, on (instrumental goals to achieve) well-being is lacking. We previously reported that the social environment is related to the well-being of community-dwelling older adults in Rotterdam in cross-sectional analysis [9]. The present study showed that social cohesion and belonging are related to the social and physical well-being of community-dwelling older adults in the Netherlands measured longitudinally. Levels of social cohesion, belonging, and realization of instrumental goals to achieve well-being varied greatly among neighborhoods. More importantly, analyses that controlled for baseline well-being and background characteristics showed that social cohesion, belonging, and changes therein predicted the well-being of these individuals.

Neighborhood social cohesion and belonging may enhance older adults’ well-being through a greater degree of social organization, including instrumental support of neighbors (especially those who are elderly, frail, and/or ill) in the form of help with basic household tasks and transportation. Such apparently minor assistance may alleviate older adults’ concerns about the future, as they can depend on neighbors' help, thereby improving well-being outcomes [6]; similarly, the ability to depend on such support may help to reduce the negative effects of increasing losses and declining gains (which accompany the aging process) on well-being [37]. Furthermore, social cohesion and belonging in neighborhoods may support older people’s social engagement in their communities, whereas poor social connections and few social ties may lead to infrequent participation and social disengagement among older citizens. Poor neighborhood conditions can result in fewer opportunities for contact and support [15].

Many neighborhoods are characterized by low levels of social cohesion and belonging; their decline and inability to achieve renewal are the products and causes of the lack of qualities such as self-help ability, mutuality, and trust [26]. Informal social networks have become increasingly important for community-dwelling older adults in the context of an aging society with growing financial constraints. As shrinking social networks have led to increased demands for care and support among elderly individuals, the monitoring of these networks and provision of other forms of support, such as neighborhood or welfare services, are important [2,38]. By distinguishing different levels of goals with regard to well-being, this study aimed to trace the consequences of living in socially deprived neighborhoods for the well-being of community-dwelling older people, and thereby to determine what changes are needed to protect their well-being. Low scores for instrumental goals, especially those related to social well-being, clearly indicate that the maintenance of overall well-being is difficult for community-dwelling older adults. In order to improve well-being in aging societies, policymakers and governments should invest in these amendable neighborhood social environments through measures aiming to improve social cohesion and belonging, or at least to avoid damaging existing networks, as such efforts will improve well-being in aging societies. The majority of current policies aim to discourage the use of expensive long-term care facilities or hospital care by augmenting informal and home care and shifting care traditionally provided in hospitals to the primary care setting, which has become the main context for the support of older people’s physical needs. This situation was observed, for example, in a nationwide study of disease management programs, which focused mainly on physical functioning, disease limitations, and lifestyle behaviors instead of investing in broader instrumental goals to maintain social as well as physical well-being [2]. Steverink [28] found that loss of comfort and affection (among the five instrumental goals to maintain well-being) were the main predictors of dependent living. Policy measures that concentrate only on physical instrumental goals (e.g., comfort) with the aim of avoiding institutionalization are inadequate, as older people’s social goals (e.g., affection) must also be supported.

Steverink [28] showed that people increasingly rely on goals that are less dependent on work, social roles, and good health (i.e., comfort and affection) for the realization of well-being as they age. The relative difficulty of realizing status, stimulation, and behavioral confirmation manifests earlier in the life span (among young-old individuals). Our study findings support this notion by showing that behavioral confirmation and stimulation deteriorated significantly over time and that status level was dramatically low at both timepoints in our sample of adults aged ≥ 70 years, especially among those living in socially deprived areas. Compensating for these losses may be particularly difficult, as resources for affection are difficult to establish. Policy makers’ attention to instrumental goals for the achievement of social as well as physical well-being is thus needed. To promote aging in place for community-dwelling older people living in socially deprived neighborhoods, protection against further deterioration of their social and physical well-being, especially regarding affection and comfort, is crucial. Integrated network approaches that aim to use available neighborhood social resources effectively and increase responsiveness to community-dwelling older people’s social and physical needs may be a means of supporting aging in place [30,39,40].

Notably, univariate analyses revealed that female gender was positively associated with well-being, whereas multivariate analyses indicated that male gender positively predicted the well-being of community-dwelling older adults in this study. These findings may be explained by men’s access to more favorable resources; older men report higher educational levels and better occupational backgrounds and are less often single than older women. In addition, older men are known to be more actively engaged in the community and to more often provide informal help to neighbors, whereas older women tend to restrict productive activities to the private domain of housekeeping [41]. Although older women may report higher levels of well-being than men, this resource advantage is likely to protect men from further deterioration of well-being and help them to better manage age-related decline and frailty.

This research is not without limitations. Our results establish the existence of significant longitudinal relationships between well-being and social cohesion and belonging among older adults, an important step prompting further studies to identify policies needed to improve these neighborhood characteristics. Respondents who completed questionnaires at T0 and T1 were on average younger, reported better well-being, were more often born in the Netherlands and higher educated compared to those who completed only one questionnaire, which may have resulted in non-response bias resulting in a possible under-estimation of the effect of social cohesion on well-being. It would be worthwhile to increase the number of attempts to visit people in their homes, since research showed that this strategy especially increases response rates among immigrant elderly [42]. While the response rate was quite high, well-being may have been higher as compared to older adults not responding at all, which may limit generalizability of our study findings. Furthermore, our study area was restricted to Rotterdam, the Netherlands, limiting the applicability of our findings to other areas. However, our findings may apply to many areas with similar neighborhood characteristics. To our knowledge, this study is the first to investigate longitudinal relationships between social cohesion and belonging and well-being among community-dwelling older adults. Thus, our results must be confirmed in further studies, especially those conducted in similar cities.

## Conclusion

In summary, this study showed that social cohesion, belonging, and changes therein predict the well-being of community-dwelling older people in the Netherlands over time. Furthermore, we found that protection against further deterioration of social and physical well-being is especially important in this population. These findings are important in the context of aging populations. Investment in neighborhood social environments is expected to benefit well-being among older citizens, especially as dependence on these environments increases with age. The strengthening of relationships among neighbors and creation of a sense of belonging are needed to improve well-being, especially among older adults.

## Appendix

### The 15-item version of the social production function instrument for the level of well-being

I will ask you a number of questions about how you feel. These questions refer to the past 3 months. For your answer, will you please choose between NEVER, SOMETIMES, OFTEN or ALWAYS? Use whichever answer is CLOSEST to the way you feel, NEVER, SOMETIMES, OFTEN or ALWAYS.

Affection

1. Do people pay attention to you?
2. Do people help you if you have a problem?
3. Do you feel that people really love you?

Behavioral confirmation

4. There are situations in which we deal with groups of people, for example at home, a club/society, or church. Do others appreciate your role in the group?
5. Do people find you reliable?
6. Do you feel useful to others?

Status

7. Do people think you do better than others?
8. Do people find you to be an influential person?
9. Are you known for the things you have accomplished?

Comfort

In the past few months, have you felt:

10. . . . relaxed?
11. . . . in good health?
12. . . . physically comfortable?

Stimulation

13. Are your activities challenging to you?
14. Do you really enjoy your activities?
15. How often are you fully concentrated when doing something?
